# Immune cell constitution in bone marrow microenvironment predicts outcome in adult ALL

**DOI:** 10.1038/s41375-018-0360-1

**Published:** 2019-01-11

**Authors:** Helena Hohtari, Oscar Brück, Sami Blom, Riku Turkki, Marjatta Sinisalo, Panu E. Kovanen, Olli Kallioniemi, Teijo Pellinen, Kimmo Porkka, Satu Mustjoki

**Affiliations:** 1 Hematology Research Unit Helsinki, University of Helsinki, Helsinki, Finland; 2 Department of Hematology, Helsinki University Hospital Comprehensive Cancer Center, Helsinki, Finland; 3 Institute for Molecular Medicine Finland, University of Helsinki, Helsinki, Finland; 4 Department of Internal Medicine, Tampere University Hospital, Tampere, Finland; 5 Department of Pathology, University of Helsinki, Helsinki, Finland; 6 HUSLAB, Helsinki University Hospital, Helsinki, Finland; 7 Department of Oncology and Pathology, Science for Life Laboratory, Karolinska Institutet, Solna, Sweden; 8 Department of Clinical Chemistry, University of Helsinki, Helsinki, Finland

**Keywords:** Cancer microenvironment, Tumour immunology, Acute lymphocytic leukaemia

## Abstract

As novel immunological treatments are gaining a foothold in the treatment of acute lymphoblastic leukemia (ALL), it is elemental to examine ALL immunobiology in more detail. We used multiplexed immunohistochemistry (mIHC) to study the immune contexture in adult precursor B cell ALL bone marrow (BM). In addition, we developed a multivariate risk prediction model that stratified a poor survival group based on clinical parameters and mIHC data. We analyzed BM biopsy samples of ALL patients (*n* = 52) and healthy controls (*n* = 14) using mIHC with 30 different immunophenotype markers and computerized image analysis. In ALL BM, the proportions of M1-like macrophages, granzyme B+CD57+CD8+ T cells, and CD27+ T cells were decreased, whereas the proportions of myeloid-derived suppressor cells and M2-like macrophages were increased. Also, the expression of checkpoint molecules PD1 and CTLA4 was elevated. In the multivariate model, age, platelet count, and the proportion of PD1+TIM3+ double-positive CD4+ T cells differentiated a poor survival group. These results were validated by flow cytometry in a separate cohort (*n* = 31). In conclusion, the immune cell contexture in ALL BM differs from healthy controls. CD4+PD1+TIM3+ T cells were independent predictors of poor outcome in our multivariate risk model, suggesting that PD1 might serve as an attractive immuno-oncological target in B-ALL.

## Introduction

Acute lymphoblastic leukemia (ALL) is a malignant disease of the early lymphoid precursors, which occurs in all age groups. The contemporary survival rates in children are excellent, whereas the treatment results in adults are still suboptimal and most patients die of their leukemia. Current treatment consists of multiagent chemotherapy to induce and consolidate remission, followed by prolonged maintenance therapy [1, 2]. Treatment guided by sensitive monitoring of minimal residual disease (MRD) and the introduction of pediatric-modeled regimens in younger adults have improved patient outcome [3–5].

Philadelphia chromosome-positive (Ph+) ALL forms the largest subgroup in adult ALL [6]. Introducing tyrosine kinase inhibitors (TKIs) into treatment regimens has improved survival in Ph^+^ ALL [7–9]. In addition to direct oncokinase inhibition, TKI therapy modulates the immune system, which may play a critical role in suppressing the growth of leukemic cells [10–14].

In addition to the anti-CD20 antibody rituximab, novel immunotherapeutic approaches such as anti-CD3-CD19 bispecific blinatumomab, chemotherapy-conjugated anti-CD22 antibody inotuzumab ozogamicin, and CD19-specific chimeric antigen receptor (CAR) T cell therapy have shown promising results [15–18]. Despite recent advances in the treatment, considerable number of ALL patients still experience relapse and leukemia-related death.

In solid tumors, the immune contexture has been shown to impact outcome [19]. In ALL, the immunological status of the bone marrow (BM) niche has not been thoroughly studied, even though increasing evidence suggests that the immune system contributes both to the development and outcome of leukemia [20, 21]. Given the success of novel immunotherapies in oncology, unveiling the immunological basis of ALL is warranted.

**Fig. 1:**
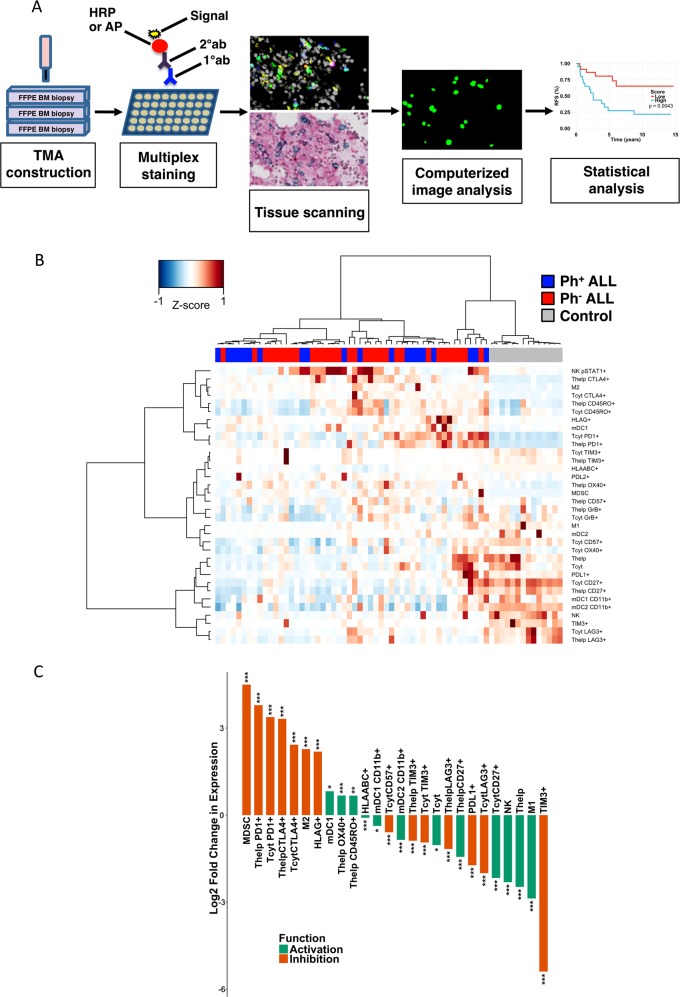
**a** Overview of the tissue microarray (TMA) analysis pipeline. TMAs were constructed from duplicate bone marrow (BM) biopsy punches from regions with high leukemic cell infiltration. TMA slides were then stained with multiplexed immunohistochemistry (mIHC) consisting of 5-plex fluorescent and 3-plex chromogenic dyes. Histological images were scanned and registered, and then cells were segmented based on differential spatial intensity using Otsu’s thresholding method. The intensity of each marker was quantified and classified. Finally, expression profiles of various markers were aggregated for more detailed data analysis. FFPE formalin-fixed paraffin-embedded. **b** Heatmap visualization of quantified immune cells (proportion of all cells) and their immunophenotypes (proportion of the parent immune cell). Spearman correlation distance and Ward linkage (ward.D2) method were used for hierarchical clustering. **c** ALL-to-control ratios were transformed by two-fold logarithmic transformation and annotated according to literature as anticancer immunity (green) or immunosuppression marker (orange). Only significantly varying (*q* < 0.05; Benjamini–Hochberg-adjusted Mann–Whitney *U* test) median values are included. It should be noted that, in case of particularly low cell numbers, this representation may skew the output, and the color annotations are a simplification of a much more diverse reality. For example, the absolute count of TIM3-expressing T cells was low both in ALL patients and controls (Fig. 2c), but the proportional difference was almost six-fold

In this study, we present a detailed description of immune cell constitution in adult precursor B cell ALL BM microenvironment at diagnosis using multiplex immunohistochemistry (mIHC) and computerized image analysis (Fig. 1a). The quantitated immune cell subsets were correlated with clinical parameters, stratifying a poor outcome group with increased number of CD4+PD1+TIM3+ T cells, higher age, and a low peripheral blood (PB) platelet count at diagnosis. These findings were validated in a separate cohort using multicolor flow cytometry (FC). Together, these results shed light on the immunological composition prevailing in ALL BM and its clinical significance.

## Materials and methods

### Study design

#### Discovery cohort

To study immune cell constitution in ALL using mIHC, we collected deposited, diagnostic-phase, formalin-fixed, and paraffin-embedded (FFPE) BM biopsies of adult precursor B cell ALL patients (*n* = 52). The cohort was retrospective and included both Ph^+^ (*n* = 31) and Ph^−^ (*n* = 21) patients. Five patients were treated in Tampere University Hospital (Finland) and the others in Helsinki University Hospital (HUH; Finland). Patients with a previously treated malignancy were excluded. BM biopsies from healthy controls (*n* = 14) were used as a reference. Control patients were referred to the hematology or internal medicine outpatient clinic most commonly due to unclear thrombocytosis or anemia, but neither hematologic malignancy, chronic infection, nor autoimmune disease was found in diagnostic examinations and 6-year follow-up (Supplementary Table S1). The patients signed a written informed consent for the study and for collection of clinical data to the Finnish Hematology Registry (FHR). The study was conducted in accordance with the Declaration of Helsinki and the HUH Ethical Committee (DNRO 303/13/03/01/2011).

#### Validation cohort

To validate the prognostic biomarkers that were found in mIHC, we used FC to analyze BM samples from 31 precursor B cell ALL patients treated in all university hospitals across Finland. The cohort was retrospective and included both Ph^+^ (*n* = 13) and Ph^−^ (*n* = 18) patients. The samples were viably frozen diagnostic-phase BM mononuclear cells obtained from the Finnish Hematology Registry and Clinical Biobank (FHRB, fhrb.fi).

#### Clinical data

As seven patients had samples in both cohorts, we studied in total 76 ALL patients. FHR served as a source for attaining patient-related clinical data. In both cohorts, we assessed altogether 33 clinical variables, including baseline laboratory values, MRD status at different time points, CD20-positivity, spleen size, comorbidities, performance score, and status of allogeneic hematopoietic stem cell transplantation (alloHSCT) (Supplementary Table S2). The observation time started from the day of diagnosis and ended when an event (relapse or death due to any cause) occurred or, in the absence of an event, at the last day of follow-up.

In the Discovery cohort, six Ph^+^ ALL patients were treated prior to the TKI era and were excluded from the survival analysis. One Ph^−^ ALL patient had received non-protocol treatment and another was treated with an older protocol and were therefore excluded from the survival analysis. All patients (*n* = 44) in the survival analysis were treated according to Finnish Leukemia Group (FLG) ALL2000 protocol or NOPHO ALL-2008 protocol [3]. In addition, all Ph^+^ patients (*n* = 25) received TKIs as part of their treatment protocol. The patients in the Validation cohort received treatments according to the same protocols as the Discovery cohort.

#### Patient characteristics

A detailed list of patient characteristics is found in Table 1. The patients in different cohorts did not differ significantly in terms of age, diagnostic-phase laboratory values, performance score, or alloHSCT prevalence (Mann–Whitney *U* test for continuous and Fisher’s exact test for categorical variables). There were slightly more females in the Discovery cohort than in the Validation cohort (45% vs. 35%). Healthy controls did not differ significantly from the Discovery cohort in terms of age or gender distribution.

**Table 1:** Patient characteristics of the Discovery (mIHC) and Validation (FC) cohort subjects included in survival analyses

Variable	mIHC (*n* = 44)	FC (*n* = 31)
Gender, female (%)	45	35
Gender, male (%)	55	65
AlloHSCT (%)	55	52
Diagnostic data (median, range)		
Age (years)	47 (16–72)	43 (19–69)
Ph^+^ (%)	57	42
CD20+ (%)	36	58
Leukocytes (10E9/l)	15.6 (0.4–174)	18.4 (0.9–188.5)
Platelets (10E9/l)	47 (3–233)	45 (3–252)
BM blasts (%)	90 (50–100)	90 (50–100)
WHO^a^ ≥1 (%)	66	71

There were no significant differences between the cohorts (Mann–Whitney *U* test for continuous and Fisher’s exact test for categorical variables). The lowest pretreatment platelet count ±2 days around the diagnosis date was selected

*alloHSCT* allogeneic hematopoietic stem cell transplantation, *BM* bone marrow, *mIHC* multiplexed immunohistochemistry, *FC* flow cytometry

^a^WHO/ECOG performance scale

### Methods

#### Tissue microarrays (TMAs)

An experienced hematopathologist evaluated the FFPE BM biopsies marking out the most representative areas with high leukemic cell infiltration. Duplicate 1 mm diameter spots were taken from the selected areas for TMA construction. Control spots from non-ALL patients were chosen from tissue regions with high cellularity.

#### Multiplexed immunohistochemistry

The TMA sections were stained with both 5-plex fluorescent and subsequent 3-plex chromogenic staining. Immune cell panels included antibodies to detect B and T lymphoid cells, natural killer (NK) and dendritic cells (DCs), macrophages, and myeloid-derived suppressor cells (MDSCs) (Supplementary Table S3). In addition, clinically relevant immune checkpoint receptors (PD1, LAG3, OX40, TIM3, CTLA4, HLA-ABC) and ligands (PD-L1, PD-L2, HLA-G) alongside with various activation markers were analyzed. The original protocol is described in detail by Blom et al. and adapted by Brück et al. [22, 23]. For antibodies, see supplementary Table S4.

#### Image preprocessing

The individual chromogen staining signals were separated by deconvolving the brightfield images [24]. Spot images were then registered with two-dimensional phase correlation method using mean image of both fluorescent and brightfield channels [25]. Before registration, mean images were downsized by a factor of eight and image histograms were adapted to each other. Image preprocessing was performed in a numerical computing platform (MATLAB, MathWorks, Natick, MA, US).

#### Image analysis

Gray-scale image channels of each TMA spot were evaluated in order to ensure the staining quality. Blurred focusing or unsuccessful image registration led to image disqualification. Unsuccessful registration was mostly induced by air bubbles in mounting media or shattered tissue. We segmented cell masks with parent immune cell markers (e.g., CD3 for T cells) using Otsu’s thresholding method and separated single cells from aggregates using intracellular intensity patterns. Cell segmentation, intensity measurements, and cell classification were implemented in an image analysis platform (CellProfiler 2.1.2 [26–28]). Total cell number for each TMA spot was calculated with Fiji from the total area of binary 4,6-diamidino-2-phenylindole images. Single-cell analysis (FlowJo v10; SI) was used for marker co-localization and cell classification with integrated intensity.

TMA spots with <1000 cells were excluded. In order to avoid bias due to cell number variation between spots, each immune cell type was quantified either as a proportion of all cells in each TMA spot or as a proportion of a defined immunophenotype to the particular cell type (e.g., CD3+CD4+/PD1+TIM3+ T cells of all CD3+CD4+ T cells [%]). The mean values of each cell class or immunophenotype were calculated from the duplicate spots obtained from the same BM sample.

#### Flow cytometry

Viably frozen BM mononuclear cells (*N* = 31) were first thawed and stained using CD3-PerCP-Cy5.5 (SK7, BD Pharmingen), CD8-FITC (SK1, BD Pharmingen), CD4-BV510 (SK3, BD Pharmingen), TIM3-PE-Cy7 (F38-2E2, Invitrogen), PD1-AlexaF647 (EH12.1, BD Pharmingen), CD45-BV421 (HI30, BD Horizon), CCR7-PE (150503, R&D Systems), and CD45RA-AlexaF700 (HI100, BD Pharmingen) antibodies. Fluorescence was measured with FACSVerse (BD Pharmingen). The proportion of CD3+CD4+/PD1+TIM3+ cells was used in survival analysis similarly as with mIHC data (Supplementary Figure S1).

#### Statistical analysis

The mIHC stainings were executed in two separate batches. In order to eliminate a batch effect, data were mean-centered. Mann–Whitney *U* test was used for comparing two groups of continuous variables. For multiple test correction, Benjamini–Hochberg’s method was applied [29]. To examine associations between survival, clinical parameters, and mIHC results, all variables with *P* < 0.20 (log-rank test, supplementary Table S5) in univariate Cox proportional hazards analysis were included in a L1-penalized elastic net regression analysis that performs both model shrinkage and variable selection [30]. The shrinkage parameter lambda (*λ*) was defined by minimum mean cross-validated error. Scaled Schoenfeld residuals were used to confirm the proportional hazards assumption of the model. In addition, competing risks were analyzed with Gray’s test [31]. We assessed model prediction by comparing the area under the receiver operating characteristic (AUROC) curves (bootstrap method, number of iterations: 4000), time-dependent ROC curves (IPCW [inverse probability of censoring weighting] approach), and *C*-statistic values of different models. Statistical analyses were performed with R v3.3.3 [32] and Prism v7.0 (GraphPad Software Inc). R packages glmnet, corrplot, survminer, survival, ggplot2, cmprsk, pROC, plotROC, timeROC, and gplots were used for statistical analysis and data visualization.

## Results

### Immune cell composition in ALL BM microenvironment differs from healthy

In order to visualize immune profiles of ALL patients (*n* = 52) and controls (*n* = 14) analyzed with mIHC (Discovery cohort), we mapped quantified immune cells and their single-marker phenotypes (Fig. 1b). Hierarchical clustering analysis revealed that immune cell subtypes linked with cytolytic activity (e.g. NK cells and granzyme B-positive T cells) were decreased and immunoregulatory markers (e.g., CTLA4+ T cells and MDSCs) increased in ALL BM, grouping ALL patients and controls distinctly from each other. The immune contexture of Ph^+^ ALL BM did not differ from Ph^−^ ALL BM.

Next we assessed significant (Mann–Whitney *U* test, *q* < 0.05) differences between immune cell subsets and their phenotypes in ALL vs. control BM. Immune cell subtypes and markers associated with regulation of immune responses (e.g., MDSCs, PD1, and CTLA4) were elevated in ALL BM, whereas pro-inflammation-related markers (e.g., M1 macrophages, NK cells, and CD27+ T cells) were decreased (Fig. 1c).

### ALL BM is reflected by diminished proportion of activated T cells and M1 macrophages, as well as lowered cytolytic activity

**Fig. 2:**
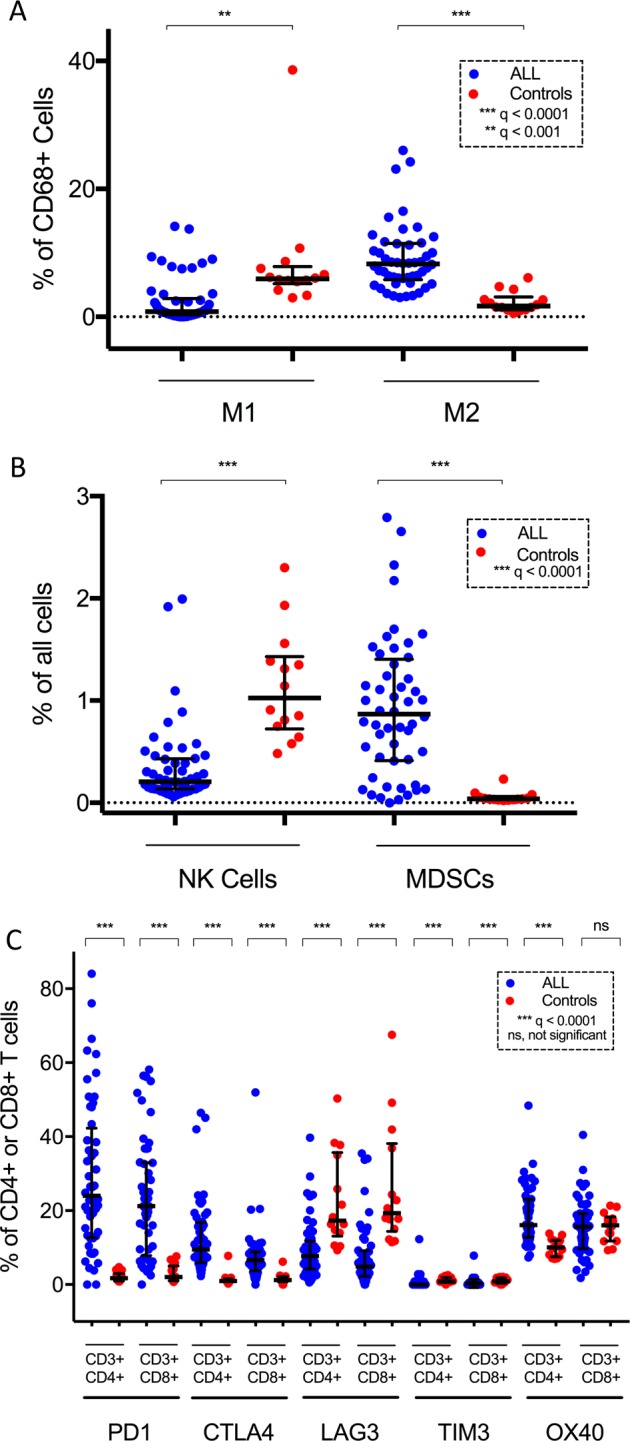
Levels of **a** M1-like and M2-like macrophages; **b** Natural killer (NK) cells and myeloid-derived suppressor cells (MDSCs); **c** PD1-, TIM3-, CTLA4-, LAG3-, and OX40-expressing T cells in multiplexed immunohistochemical analysis were compared with Mann–Whitney *U* test and *p* values adjusted using Benjamini–Hochberg method (*q*-values). **q < 0.001, ***q < 0.0001

A T helper type 1 (Th1)-driven immune response and especially CD8+ T cells have been associated with beneficial prognosis and anticancer protection [33]. In addition, M1-like macrophages secrete high levels of inflammatory cytokines and are associated with antitumor responses [34]. Therefore, we investigated whether the levels of CD8+ T cells, NK cells, and M1-polarized macrophages are affected in ALL patients. The proportion of M1-like macrophages was decreased (0.8% vs. 5.9% of CD68+ cells in ALL compared to healthy BM, *q* = 0.0002; Fig. 2a). Similarly, the proportion of CD8+granzyme B+CD57+ T cells (11.5% vs. 24.0% of CD8+ T cells, *q* = 0.0001) as well as CD27+ T cells (7.9% vs. 21.5% of CD3+CD4+ T cells, *q* < 0.0001 and 7.7% vs. 34.6% of CD3+CD8+ T cells, *q* < 0.0001; Supplementary Figure S2) was decreased in ALL BM, suggesting suppressed cytolytic and co-stimulation ability, respectively. The proportion of NK cells of all cells (0.2% vs. 0.9%, *q* < 0.0001, Fig. 2b) was decreased in comparison to controls.

### Increased levels of myeloid M2-polarized macrophages and MDSCs in ALL BM

As M2-like macrophages and MDSCs are able to promote tumor growth by dampening Th1-mediated immune responses, we next examined the level of immunosuppressive myeloid cells [34, 35]. M2-like macrophages were enriched in ALL BM (8.3 vs. 1.7%, of CD68+ cells, *q* < 0.0001; Fig. 2a). Similarly, the proportion of MDSCs was increased (0.9% vs. 0.04% of all cells, *q* < 0.0001; Fig. 2b).

### The expression of immune checkpoints PD1 and CTLA4 is upregulated in T cells

Next, we evaluated the expression of pivotal immune checkpoint molecules in T cells (Fig. 2c). The expression of PD1 (24.0 vs. 1.7% of CD4+ cells, *q* < 0.0001 and 21.2 vs. 2.0% of CD8 cells, *q* < 0.0001) and CTLA4 (9.4 vs. 0.9% of CD4+ cells, *q* < 0.0001 and 6.6 vs. 1.2% of CD8+ cells, *q* < 0.0001) was pronounced in ALL BM. However, the expression of LAG3 (7.7 vs. 17.3% of CD4+ cells, *q* < 0.0001 and 4.8 vs. 19.3% of CD8+ cells, *q* < 0.0001) and TIM3 (0.0 vs. 0.8% of CD4+ cells, *q* < 0.0001 and 0.0 vs. 0.9% of CD8+ cells, *q* < 0.0001) were decreased in ALL compared to the control BM. The expression of OX40 was increased in CD4+ T cells (16.1 vs. 10.1% of CD4+ cells, *q* < 0.0001), but no difference was seen in CD8+ T cells.

### Antigen-presenting myeloid DC type 1 (mDC1) and CD4-positive memory T cells are increased in ALL BM

T cell priming is dependent on antigen presentation. mDC1 are essential in presenting cancer-related antigens and inducing a Th1-fashioned immune response by producing high amounts of interleukin-12 [36]. The proportion of mDC1s was enriched (3.5% [interquartile range (IQR) 1.7–5.3%] vs. 2.0% [IQR 1.1–2.8%] of all cells, *q* = 0.03). In addition, the proportion of CD4+CD45RO+ memory T cells (41.0% [IQR 30.5–51.4%] vs. 25.8% [IQR 19.1–30.1%] of CD4+ cells, *q* = 0.002) was elevated. The percentage of CD8+CD45RO+ T cells was increased as well but did not reach statistical significance.

### CD4+PD1+TIM3+ T cells, platelet count, and age predict survival

To study the synergy and translational significance of immune profiles and clinical parameters, we performed L1-penalized Cox regression analysis on preselected covariates (*P* < 0.20 in univariate Cox regression). The resulting risk model was used to stratify patients into dichotomous, equally sized groups (i.e., high-risk and low-risk groups). The primary endpoint was relapse-free survival (RFS).

**Fig. 3:**
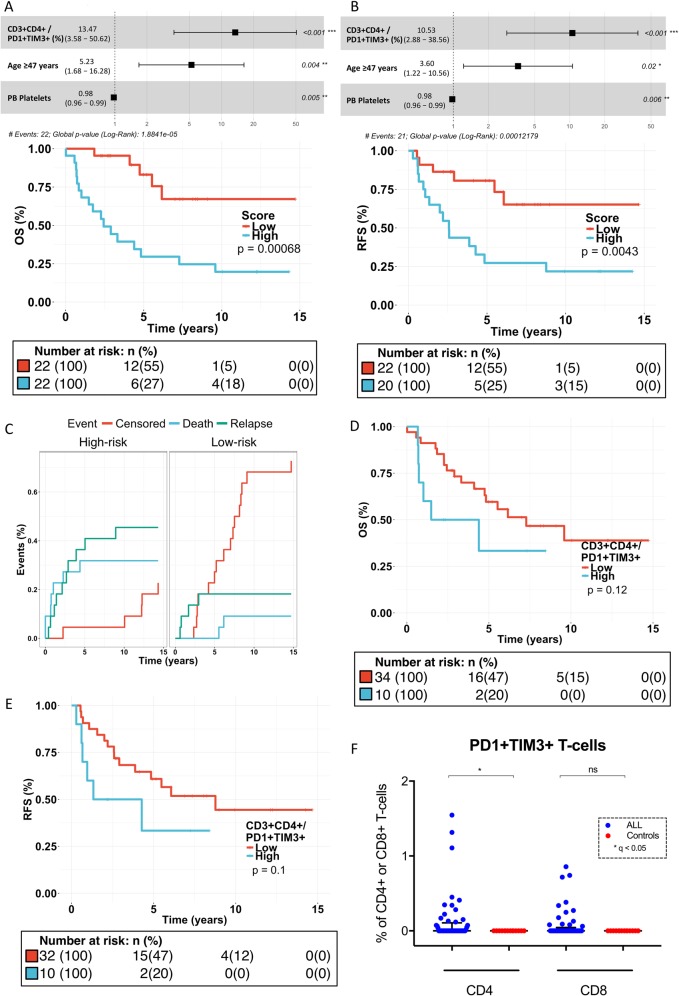
Survival analysis in the Discovery cohort (multiplex immunohistochemistry). Forest plot of the risk stratification model for **a** OS (overall survival) and **b** RFS (relapse-free survival). The risk stratification model was divided by median into high- and low-risk groups and plotted for **a** OS and **b** RFS (Cox regression analysis, log-rank test). **c** Competing risk analysis of EFS was performed for the high- and low-risk group stratification model (Gray’s test). **d** OS and **e** RFS curves (Cox regression analysis, log-rank test) for patients categorized by PD1+TIM3+ high-expressing (cutoff > 0.1% CD4+PD1+TIM3+ T cells) and low-expressing groups. **f** Levels of PD1+TIM3+ double-positive T cells in ALL and healthy control bone marrow. *q < 0.05

In the risk stratification model, high expression of BM CD4+PD1+TIM3+ T cells, age above the cohort median, and low PB platelet count at diagnosis differentiated a poor survival group. Furthermore, multiple other potential immune biomarkers were discovered in univariate analysis, even though these did not stand out in our multivariate model (Supplementary Table S5). The hazard ratio (HR) for overall survival (OS) in the high-risk group was 4.9 (95% confidence interval (CI) 1.8–13.3; *P* = 0.0007, log-rank test; Fig. 3a), for RFS 3.7 (95% CI 1.4–9.6; *P* = 0.004, log-rank-test; Fig. 3b), and for event-free survival (EFS) 4.0 (95% CI 1.6–10.3; *P* = 0.002; Supplementary Figure S3A,B). The competing risk analysis demonstrated that both deaths (*P* = 0.052, Gray’s test) and relapses (*P* = 0.072) were more prevalent and censoring less prevalent (*P* = 0.0058) in high-risk patients (Fig. 3c). In univariate analysis, the high expression (>0.1% of T cells) of PD1+TIM3+ double-positive CD4+ T cells trended toward poor survival (Fig. 3d, e; Supplementary Figure S3C), and relapses were more common in high-expressing patients in competing risk analysis (Supplementary Figure S3D). Although the mean proportion of PD1+TIM3+ T cells in ALL patients is low, it is heterogeneously expressed (mean 0.12%, range 0–1.55%, standard deviation (SD) 0.31; Fig. 3f). Patient characteristics of the high- and low-risk groups in the risk stratification model are described in Supplementary Table S6. The high-risk group was characterized with higher BM blast proportion (*P* = 0.003), but no other association with clinical parameters was found.

**Fig. 4:**
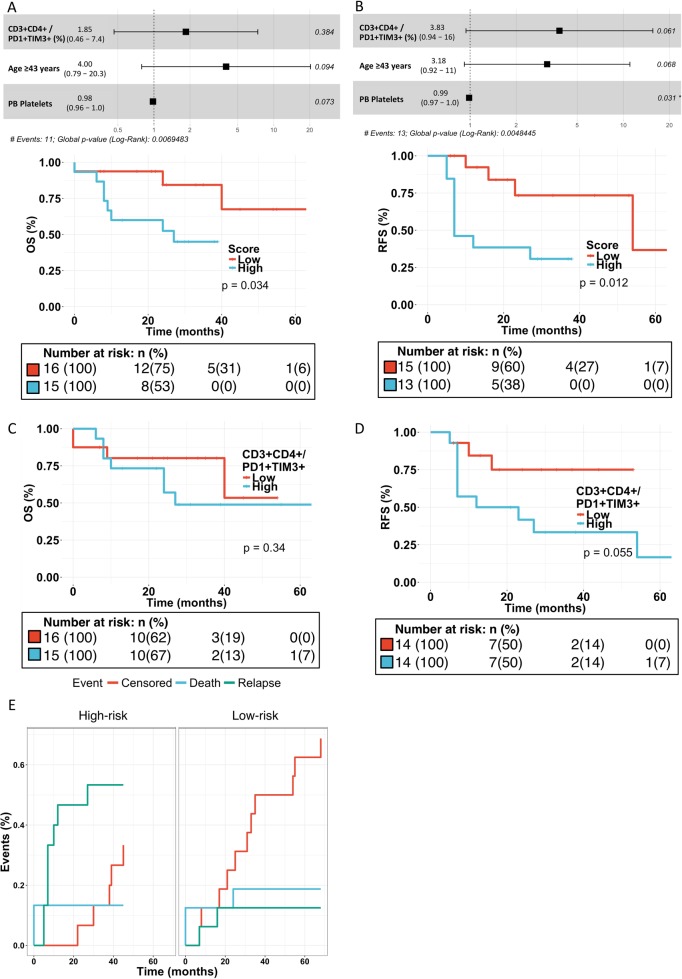
Survival analysis in the Validation cohort (flow cytometry). Forest plot of the risk stratification model for **a** OS (overall survival) and **b** RFS (relapse-free survival). The risk stratification model was divided by median into high- and low-risk groups and plotted for **a** OS and **b** RFS (Cox regression analysis, log-rank test). Survival curves of CD3+CD4+/PD1+TIM3+ cells categorized into two groups by median and plotted for **c** OS and **d** RFS. **e** Competing risk analysis of EFS was performed for the high- and low-risk group stratification model (Gray’s test)

The results were validated in the Validation cohort. Even with a sample size of 31 patients, high proportion of CD4+PD1+TIM3+ T cells (divided according to cohort median), higher age (divided according to cohort median), and low platelet count predicted poor OS (HR 4.7, 95% CI 0.98–22.5; *P* = 0.03, log-rank test; Fig. 4a), RFS (HR 4.9, 95% CI 1.3–19.0; *P* = 0.01, log-rank test; Fig. 4b), and EFS (HR 4.4, 95% CI 1.3–14.2; *P* = 0.009; Supplementary Figure S4A, B). The mean proportion of PD1+TIM3+ T cells remained small in ALL samples (mean 0.88%, range 0.00–10.11%, SD 1.88). Similarly, in univariate analysis, high proportion of CD4+PD1+TIM3+ T cells trended toward poor RFS and EFS but not OS (Fig. 4c, d; Supplementary Figure S4C). In competing risk analysis, relapses were more prevalent in high-risk patients (*P* = 0.014, Gray’s test; Fig. 4e) and censoring less common (*P* = 0.013) in patients with PD1+TIM3+ expression superior to median in CD4+ T cells (Supplementary Figure S4D). Patient characteristics of the risk groups in the Validation cohort are described in Supplementary Table S6.

### High proportion of PD1+TIM3+ T cells is associated with immunologic activity

**Fig. 5:**
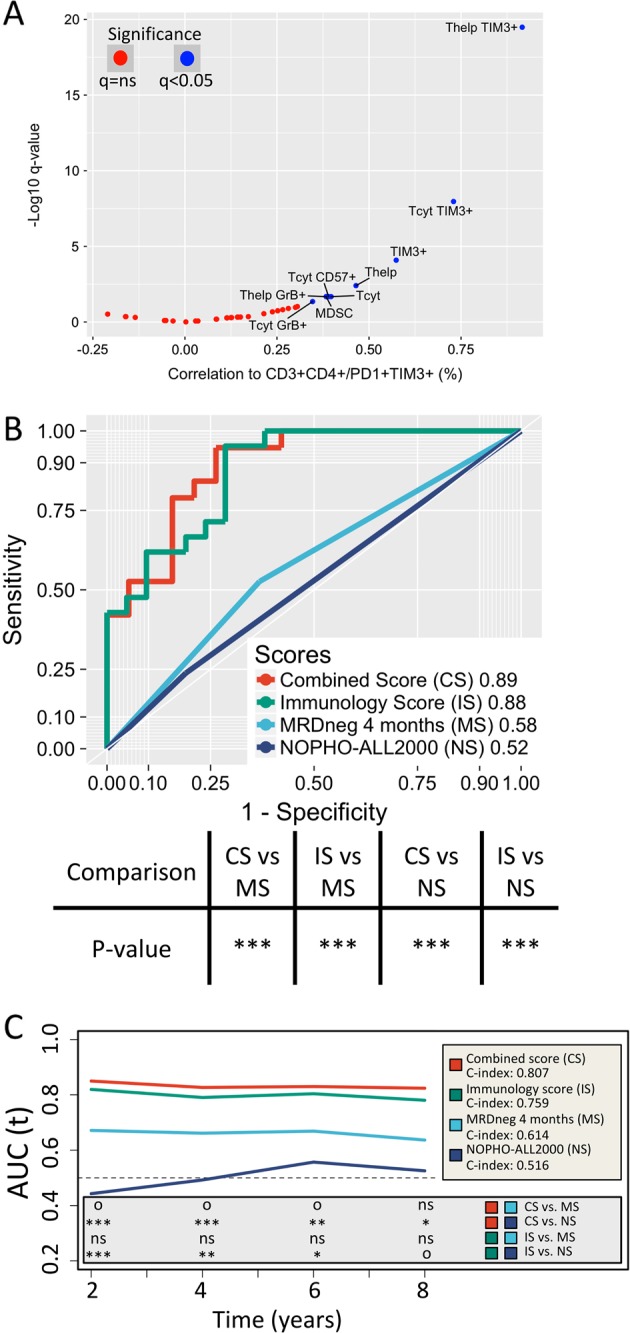
**a** The correlation between CD4+PD1+TIM3+ T cell count and other immune cell phenotypes. Significant values (*q* < 0.05, Spearman correlation) are annotated in blue. To determine the prediction power of **b** RFS, area under the receiver operating characteristic curves (AUROC) of our model (green line) was compared to stratification by MRD status at 4 months (light blue line) with the bootstrap method (number of iterations: 4000). Similarly, a combination of both models (red line) was developed and compared to stratification by MRD status at 4 months and original study protocol stratifications (dark blue line). Symbols for significance of the comparison: *P < 0.05, **P < 0.01, ***P < 0.001. **c** The time-dependent receiver operating characteristic (ROC) curves (IPCW [inverse probability of censoring weighting] approach) and *C*-statistic values for RFS. Our prognostic model (green line) is compared to stratification by MRD status at 4 months post-diagnosis (light blue line). Their combined model (red line) is compared to stratification by MRD status at 4 months post-diagnosis and original study protocol stratifications (dark blue line). Significance is indicated at 2-, 4-, 6-, and 8-year time points with following symbols: ^o^*P* < 0.10, *P < 0.05, **P < 0.01, ***P < 0.001

As the proportion of CD4+ T cells expressing PD1+TIM3+ were associated with poor survival, we next studied the interaction of CD4+PD1+TIM3+ T cells with clinical and immune parameters. There was no correlation between PD1+TIM3+ expression and clinical laboratory parameters or age (Supplementary Figure S5). Interestingly, the abundance of CD4+ and CD8+ T cells and their expression of GrB and TIM3 and the number of MDSCs correlated (Spearman correlation) positively with CD4+PD1+TIM3+ T cells (Fig. 5a).

### Integrating MRD status to the multivariate model

Achieving MRD negativity is one of the strongest predictors of outcome in adult ALL [37]. Therefore, we compared long-term survival prediction ability of our multivariate risk model to risk stratification by MRD status at 4 months post-diagnosis. MRD was primarily measured using polymerase chain reaction (PCR)-based approaches (quantitative reverse transcription PCR/allele-specific oligonucleotide-PCR), but in the absence of a suitable follow-up marker for PCR, MRD measurement using multiparameter FC with sufficient sensitivity (10^−4^) was accepted as well. MRD negativity was defined as <10^−4^ leukemic blasts per healthy cells. Our model predicted RFS, EFS, and OS with higher confidence than MRD monitoring or risk stratification according to original NOPHO-ALL2008 [3] or FLG ALL2000 protocols (Supplementary Table S7) using AUROC comparison (bootstrap method; Fig. 5b and Supplementary Figure S6A, B) and trended toward superior prediction with both *C*-statistic and time-dependent AUC comparison (IPCW approach; Fig. 5c and Supplementary Figures S6C, D). Interestingly, by supplementing MRD status at 4 months with the immunoprofiling model, each covariate remained independent (*P* < 0.05) and the overall model improved prediction. Similar model improvement did not occur if the model was combined with NOPHO-ALL and ALL2000 risk classification.

## Discussion

The results of integrative immune profiling suggest that immune cell subtypes and markers associated with immune regulation (such as MDSCs, PD1, and CTLA4) are increased in ALL BM compared to healthy controls. In addition, high proportion of CD4+PD1+TIM3+ double-positive T cells, older age, and low platelet count at diagnosis identified a group with poor survival in two separate cohorts.

mIHC allows in-depth cytometric evaluation of different immune cell subtypes in their original BM microenvironment. When analyzing samples in the TMA format, hundreds of samples and tens of different marker combinations can be analyzed simultaneously [38]. FFPE preserves samples and bypasses the possible effects of cryopreservation on cell number, viability, and phenotype [39], thus serving retrospective discovery studies ideally. Our mIHC approach was supplemented with automated image analysis for fast and objective immune cell classification and quantification. We validated prognostic biomarkers with FC, which is used in routine clinical practice for diagnostic and follow-up purposes.

In solid tumors, the immune contexture both before and during treatment has been shown to predict treatment responses to chemotherapy and immune checkpoint therapy as well as survival [40–46]. In hematological malignancies, the role of immune cell composition is still largely unrevealed, and to our knowledge, this is the first comprehensive immunological study on precursor B-ALL BM.

As the immune system of cancer patients is constituted of diverse cell populations engaging in a complex and dynamic interaction [47], we investigated a wide variety of cell populations and phenotype markers with known clinical significance or well-established role in immunology [19, 48, 49]. Our results showed that, when compared to healthy BM, in ALL, the proportion of antitumor-associated M1-like macrophages was decreased, and the proportion of protumor-associated M2-like macrophages and MDSCs was increased, consistent with previous studies [50]. Also the proportion of NK cells was lower, as has been previously described [51]. Further, antigen-presenting mDC1s and the proportion of CD4+ memory T cells were enriched in ALL BM advocating for possible augmented antigen-presentation capability.

T cells are perceived as the pivotal effector cell type in immuno-oncology and most likely also in B cell ALL owing to the success of treatments such as CD19-targeted CAR T cell therapy and T cell engaging therapies [16–18, 52, 53]. Their level of infiltration into the tumor core and invasive margin might predict, at least in colorectal cancer, survival with higher accuracy than the classical tumor–node–metastasis classification [40, 41]. Leukemia lacks an objective central tumor and invasive margin, thus making spatial immune stratification inapplicable.

Given the essential role linked to T cells, we designed the characterization panels to focus on T cells and their immunophenotype. Overall, the proportion of CD27+ T cells and CD8+granzyme B+CD57+ T cells was decreased, and the expression of immune checkpoint molecules PD1 and CTLA4 on T cells was increased. On the other hand, the expression of LAG3 and TIM3 was decreased and the expression of OX40 on CD4+ T cells increased, but not on CD8+ T cells. The reason for contradictory expression patterns of immune checkpoint molecules remains unclear but might be explained with different regulatory signaling pathways. Immune checkpoint inhibitors have gained a strong foothold especially in the treatment of metastatic melanoma and non-small-cell lung cancer. Currently limited data are available on their efficacy in ALL; however, several studies regarding the use of anti-PD1 (NCT02819804, NCT02767934) and anti-CTLA4 (NCT02879695, NCT01919619) antibodies in ALL are ongoing.

Interestingly, the high number of CD4+PD1+TIM3+ double-positive T cells, advanced age, and low platelet count at diagnosis differentiated a poor survival group in two separate cohorts. Our model outweighed stratification by MRD and the original study protocol risk classification in predicting long-term survival in our small Discovery cohort, but the combination of our model with simple MRD prediction strengthened them both. It is, however, fair to keep in mind, that there were relatively few NOPHO-ALL2008 patients, and almost all of them fell in the high-risk category, making objective assessment of these two study protocol risk stratifications in our study cohort inapplicable. While the model has to be validated in a larger cohort, the high-risk group seemed to be associated with higher BM blast proportion and PB leukocyte count, which are markers of high disease burden. High expression of CD4+PD1+TIM3+ T cells might predict poor survival in adult B cell ALL patients [54]. Interestingly, high CD4+PD1+TIM3+ T cell proportion was associated with cytolytic (T cells and GrB expression), senescent (CD57 expression), and suppressive immune subsets (MDSCs). Despite remaining unexplained by our study, PD1+TIM3+CD4+ T cells might arise from prolonged immune response against blasts, e.g., immune exhaustion, or chronic inflammation related to BM expansion [55, 56]. While these quantitative findings are merely descriptive by nature, decreased effector and increased immunoregulatory phenotype prevailing in ALL BM might reflect a switch from immune activation to immunosuppressive state. Further functional studies are warranted to address this question. In addition, as aging affects the immune system substantially [57], it would be interesting to investigate the immune microenvironment in pediatric ALL BM.

In conclusion, our results suggest that ALL BM has a unique immune cell composition that is also associated with clinical response to therapy. Additional research clarifying the impact of ALL immunobiology on disease progression and solving how to translate the immunophenotype features into treatment biomarkers remain imperative.

## Supplementary information

Supplemental material
