# Supplementary material for: Immune cell constitution in bone marrow microenvironment predicts outcome in adult ALL
Source: Leukemia. 2019 Jan 11;33(7):1570–82. doi: 10.1038/s41375-018-0360-1 (PMC6755974; doi:10.1038/s41375-018-0360-1)
Supplement: Supplementary file 1 — Supplemental material [file 41375_2018_360_MOESM1_ESM.pdf]

Immune cell constitution in bone marrow microenvironment predicts outcome in adult ALL

Helena Hohtari,[1*] Oscar Brück,[1*] Sami Blom,[2] Riku Turkki,[2] Marjatta Sinisalo,[3] Panu E. Kovanen,[4] Olli Kallioniemi,[2,5] Teijo Pellinen,[2] Kimmo Porkka,[1] and Satu Mustjoki[1,6]

[1]Hematology Research Unit Helsinki, University of Helsinki and Department of Hematology, Helsinki University Hospital Comprehensive Cancer Center, Helsinki, Finland.

[2]Institute for Molecular Medicine Finland, University of Helsinki, Helsinki, Finland.

[3]Department of Internal Medicine, Tampere University Hospital, Tampere, Finland.

[4]Department of Pathology, University of Helsinki and HUSLAB, Helsinki University Hospital, Helsinki, Finland.

[5]Science for Life Laboratory, Karolinska Institutet, Department of Oncology and Pathology, Solna, Sweden.

[6]Department of Clinical Chemistry, University of Helsinki, Helsinki, Finland.

* H.H and O.B. contributed equally to this article.

This file includes supplementary methods, reagent specification, and supplementary data supporting the original manuscript.

Supplementary Table S1. Characteristics of control subjects* and indications for bone marrow biopsy.

**Gender**	**Age**	**Indication for bone marrow biopsy**
male	61	Persistent thrombocytosis
male	56	Thrombocytosis
female	57	Mild leukopenia and thrombocytopenia
male	13	Mild neutropenia
male	60	Fluctuating leukocytosis and mild thrombocytosis
male	61	Mild thrombocytopenia
male	44	Mild eosinophilia
male	65	Unspecific, hypodense focal lesions in spleen
female	40	Thrombocytosis and leukocytosis for several months
female	25	Mild neutropenia
female	44	Moderately elevated hemoglobin and mild thrombocytosis
female	47	Iron-deficiency anemia with mild neutropenia
male	54	Persistent erythrocytosis
female	43	Mild anemia and lymphopenia

*Control subjects have not been diagnosed with any hematological or autoimmune disease and are no longer under active surveillance at the Department of Hematology or Internal Medicine.

Supplementary Table S2. Clinical parameters.

**Variable**	**Unit**
Time from treatment start to remission	days
Remission	1=yes, 0=no
TKI treatment	1=yes, 0=no
Treatment protocol (intention-to-treat)	
Gender	0 = male, 1 = female
Age at diagnosis	years
Blast, blood, proportion of leukocytes	%
Blasts, bone marrow	%
Leukocytes, blood, absolute count	E9/L
Lymphocytes, blood, absolute count	E9/L
Lymphocytes, blood, proportion of leukocytes	%
Lymphocytes, blood, absolute count	E9/L
Neutrophils, blood, absolute count	E9/L
Neutrophils, blood, proportion of leukocytes	%
Thrombocytes, blood, absolute count	E9/L
CD20-status at diagnosis	1 = positive, 0 = negative
Ph+ chromosome	1 = yes, 0 = no
BCR-ABL1 transcript type	1 = P190, 2 = P210
Spleen size	cm
Spleen size	cm over 11 cm
Smoking	1 = smoking history, 0 = no smoking history
Number of comorbidities	0 = 0, 1 = 1-2, 2 = ≥3

WHO-ECOG class	0-4
AlloHSCT	1 = yes, 0 = no
Additional cytogenetic abnormalities (Ph+ not included)	1 = yes, 0 = no
MRD status at 1 month	1 = positive, 0 = negative
MRD status at 2 months	1 = positive, 0 = negative
MRD status at 3 months	1 = positive, 0 = negative
Achieving MRD-negativity before 4 months	1 = yes, 0 = no
Cytogenetic class*	1 = favorable, 2 = intermediate/not determined, 3 = adverse
Duration of neutropenia after induction treatment, until neutrophils 0.1*E9/L	days
Duration of neutropenia after induction treatment, until neutrophils 0.5*E9/L	days
Duration of neutropenia after induction treatment, until neutrophils 1.0*E9/L	days

* Favorable = High hyperdiploidy; Adverse = Hypodiploidy, t(9;22)(q34;q11.2), t(4;11)(q21q23), or complex karyotype (≥5 abnormalities). AlloHSCT, allogeneic hematopoietic stem cell transplantation; MRD, minimal residual disease.

Supplementary Table S3. Markers used in immune cell characterization

**Cell name**	**Marker combination**
Cytotoxic T-cells	CD3+CD8+
Helper T-cells	CD3+CD4+
NK-cells	CD3-CD56+
M1 macrophages	CD3-CD68+pSTAT1+cMAF-
M2 macrophages	CD3-CD68+pSTAT1-cMAF+
MDSCs	CD11b+CD33+HLADR-
mDC1	CD11c+BDCA1+

NK-cells, natural killer cells; MDSCs, myeloid derived suppressor cells; mDC1, myeloid dendritic cells type 1

Supplementary Table S4. Antibody panel used in multiplexed immunohistochemistry.

LPR, liquid permanent red. VG, vina green. GrB, granzyme B. MDSCs, myeloid-derived suppressor cells. Details of the antibodies are published by Brück et al (Leukemia 2018, in press).

**Panels/Detection probes**	**GFP**	**Cy3**	**Cy5**	**Cy7**	**LPR**	**VG**
**T cell activity**	GrB 1:100	CD57 1:400	CD8 1:25	CD4 1:25		CD3 1:250
**Memory T-cells**	CD27 1:500	CD25 1:25	CD8 1:25	CD4 1:25		CD3 1:250
**Immune checkpoints 1**	PD-1 1:1500	TIM-3 1:2500	CD8 1:25	CD4 1:25		CD3 1:250
**Immune checkpoints 2**	LAG-3 1:150	CTLA-4 1:150	CD8 1:25	CD4 1:25		CD3 1:250
**Immune checkpoints 3**	PD-1 1:1500	OX40 1:25	CD8 1:25	CD4 1:25	CD45RO 1:250	CD3 1:250
**Cancer cell ligands**	HLA G 1:25	PD-L1 1:50	TIM-3 1:100	HLA-ABC 1:100	PD-L2 1:250	CD34 1:100
**B-cells, NK-cells and macrophages**	CD56 1:300	pSTAT1 1:100	CD3 1:25	CD20 1:25	CMAF 1:150	CD68 1:250
**Dendritic cells and MDSCs**	CD11b 1:250	CD33 1:200	BDCA-3 1:25	HLA-DR 1:200	CD11c 1:250	BDCA-1 1:100

Supplementary Table S5. Covariates used in the development of the immunohistochemistry risk stratification model (P<0.20, log-rank). Ns, not significant (P≥0.20); PB, peripheral blood.

Covariate	EFS, P-value	RFS, P-value	OS, P-value
PB Platelets	0.0090	0.0087	0.019
CD3+CD4+/PD1+TIM3+ [%]	0.013	0.013	0.0015
CD3+CD4+/TIM3+ [%]	0.021	0.021	0.0038
CD3+CD8+/TIM3+ [%]	0.025	0.024	0.0049
CD3+CD4+/PD1-TIM3+ [%]	0.029	0.028	0.0061
CD3+CD8+/PD1-TIM3+ [%]	0.031	0.031	0.0070
TIM3+ [%]	0.031	0.031	0.0072
PB neutrophils [%]	0.036	0.036	0.072
CD3+CD8+/PD1+TIM3+ [%]	0.039	0.038	0.010
CD68+CD3-/pSTAT1+cMAF- [%]	0.075	0.074	0.029
CD3-CD56+ [%/100]	0.091	0.073	0.026
Age by median (1 ≥47 years, 0 <47 years)	0.10	0.097	0.047
PDL2+ [%]	0.11	0.13	0.038
CD11c+BDCA1+ [%/100]	0.11	0.11	0.089
MRD neg at 3 months [1 = yes, 0 = no]	0.12	0.13	ns
Age	0.13	0.13	0.055
CD3+CD8+/CTLA4-LAG3+ [%]	0.13	0.13	0.090
CD3+CD8+/CTLA4+ [%]	0.14	0.13	0.11
CD3+CD8+/CD27+ [%]	0.14	0.14	0.15
CD3+CD4+/PD1+TIM3-	0.16	0.17	0.19

[%]			
CD3+CD8+/CTLA4+LAG3- [%]	0.17	0.16	0.071
CD3-CD56+/pSTAT1+ [%]	0.17	0.18	0.12
CD20	0.17	0.17	0.094
CD3+CD4+/PD1+ [%]	0.18	0.18	ns
CD3+CD8+/CTLA4+LAG3+ [%]	0.19	0.19	ns
CD11c+BDCA1+/CD11b+ [%]	0.19	0.20	ns
Size of splenomegaly [cm over 11 cm]	0.20	0.19	0.12
Spleen size (cm)	ns	ns	0.13
CD3+CD4+/PD1+OX40- [%]	ns	ns	0.18

Supplementary Table S6. Patient characteristics at diagnosis in the immunohistochemistry (IHC) and flow cytometry (FC) high and low-risk groups

**Variable**	**IHC high risk (n=22)**	**IHC low risk (n=22)**
Gender, female (%)	45	45
Gender, male (%)	55	55
AlloHSCT (%)	38	62
Age (years)*	55 (26-72)	44 (16-68)
Ph^+ (%)	56	44
KMT2A-R (%)	5	14
iAMP21 (%)	0	5
t(12;21) (%)	0	0
t(1;19) (%)	0	0
Hypodiploid karyotype (<45 chromosomes) (%)	14	5
Complex karyotype (≥5 aberrations) (%)	23	9
High hyperdiploid (51-65 chromosomes) (%)	0	0
CD20+ (%)*	55	18
White blood cells >100 10E9/l (%)	14	5
Leukocytes (10E9/l)	17.4 (0.4-174)	13.0 (1-163.4)
Platelets (10E9/l)***	36 (3-127)	83 (20-233)
BM blasts (%)**	90 (50-100)	85 (55-95)
WHO† ≥ 1 (%)	64	68

CNS1 (%)	100	100
CNS2 (%)	0	0
CNS3 (%)	0	0
**Variable**	**FC high risk (n=15)**	**FC low risk (n=16)**
Gender, female (%)	47	25
Gender, male (%)	53	75
AlloHSCT (%)	60	44
Age (years)	46 (24-68)	31 (19-69)
Ph^+ (%)	40	44
KMT2A-R (%)	0	0
iAMP21 (%)	0	0
t(12;21) (%)	0	0
t(1;19) (%)	0	0
Hypodiploid karyotype (<45 chromosomes) (%)	13	13
Complex karyotype (≥5 aberrations) (%)	20	0
High hyperdiploid (51-65 chromosomes) (%)	0	0
CD20+ (%)	47	69
White blood cells >100 10E9/l (%)	7	6
Leukocytes (10E9/l)	20.9 (0.9-188.5)	11.1 (1-124.7)

Platelets (10E9/l)***	27 (3-96)	83 (25-252)
BM blasts (%)	90 (54-100)	88 (50-95)
WHO† ≥ 1 (%)	80	63
CNS1 (%)	86	94
CNS2 (%)	7	6
CNS3 (%)	7	0

Values are presented as median (range). *P<0.05, **P<0.005, ***P<0.0005, and if not stated P≥0.05 (Mann-Whitney U test for continuous and Fisher's exact test for categorical variables) †WHO/ECOG performance scale; allogeneic hematopoietic stem cell transplantation (alloHSCT); bone marrow (BM). The lowest pretreatment platelet count ±2 days from the day of diagnosis was selected.

Supplementary Table S7. Risk stratification for B-cell ALL in FLG ALL2000 protocol (amendment 2014).

High risk

At diagnosis

- BCR-ABL1-positive disease (if MRD-positive during treatment)
- t(4;11), *MLL-AF4*
- t(17;19), *TCF3-HLF*

During the treatment

- No cytological remission after 1. induction
- Residual disease >0,01% (>1x10-4) after 2. consolidation (flow cytometry/PCR)
- Increasing levels of residual disease (1,0 log increase repeatedly within 2 weeks/ molecular relapse)

Standard risk

Other than high risk disease

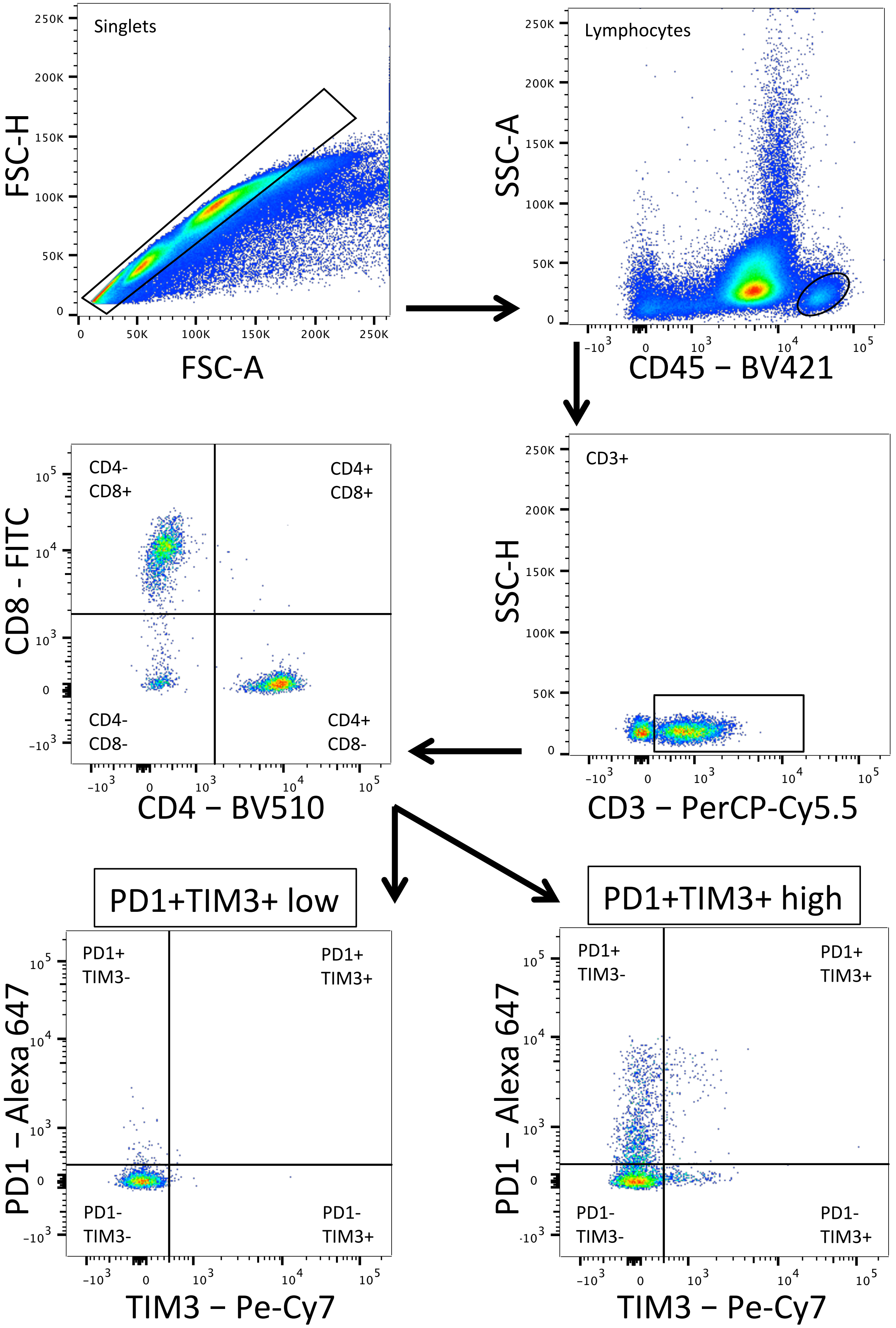

Supplementary Figure S1. Gating strategy for flow cytometry analysis to identify and quantify CD4+ and CD8+ T cells and their PD1+ and TIM3+ subsets.

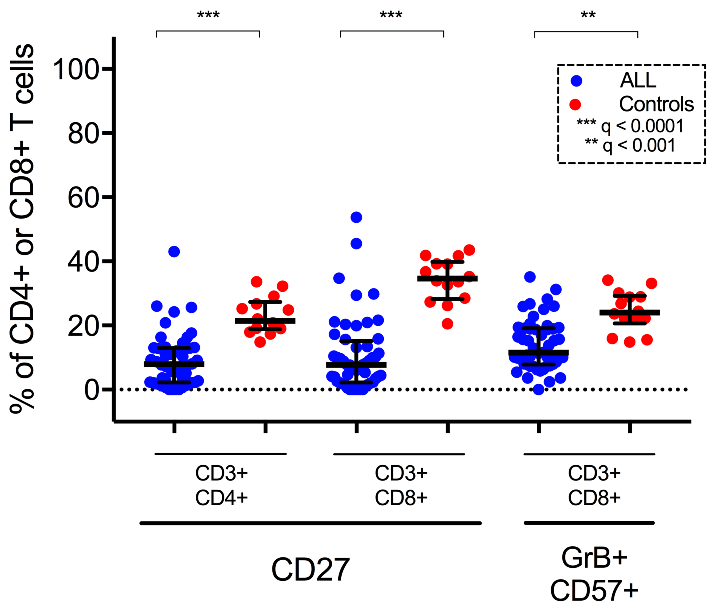

Supplementary Figure S2. Levels of CD27+ T-cells and granzyme B+ CD57+ CD8+ T-cells in multiplexed immunohistochemistry analysis were compared with Mann-Whitney U test and p-values adjusted using Benjamini-Hochberg method (q-values).

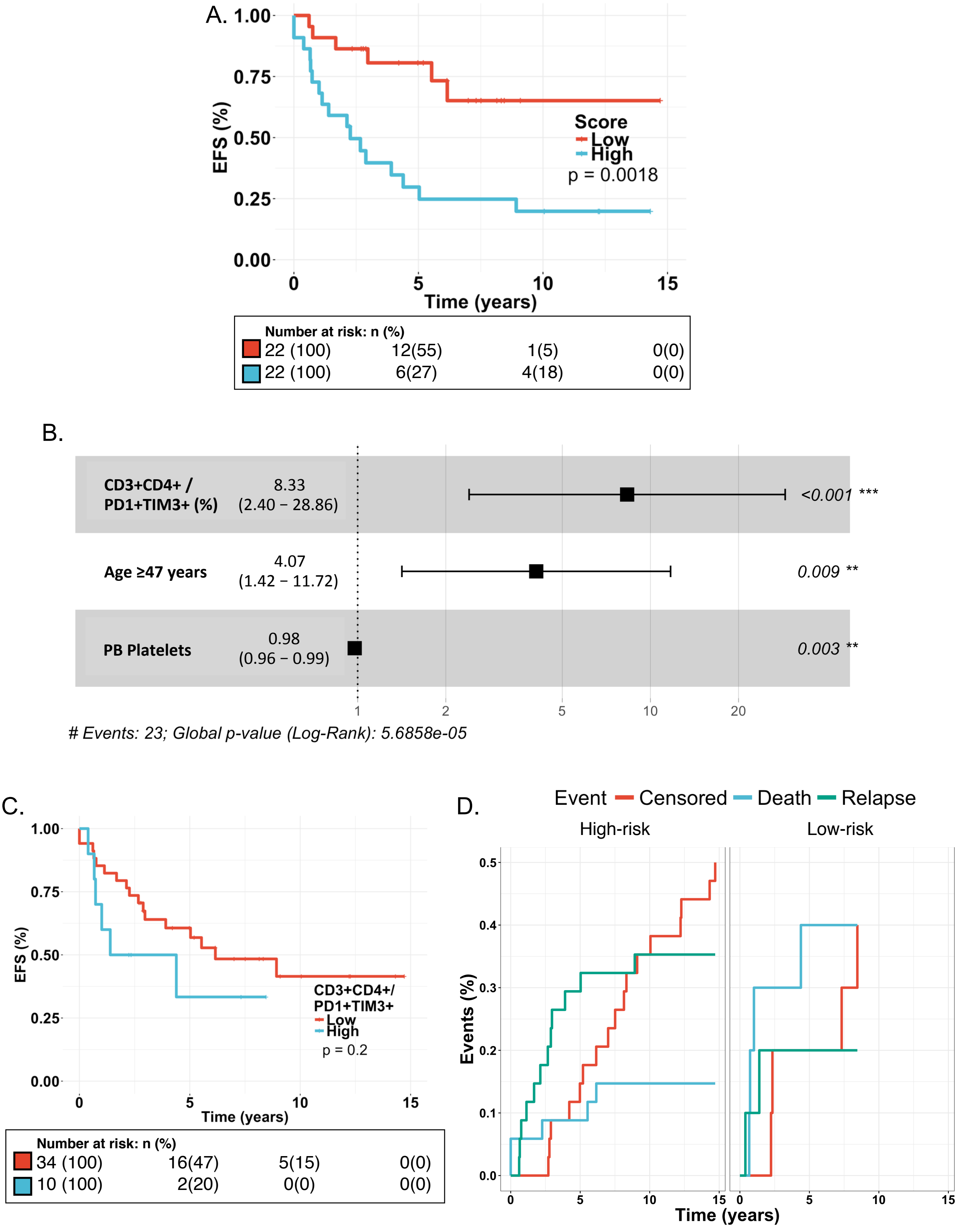

Supplementary Figure S3. Survival analysis in the Discovery cohort. (A) The risk stratification model was divided by median into high and low-risk groups and plotted for event-free survival (EFS; Cox regression analysis, log-rank test). (B) Forest plot of the risk stratification model with continuous covariates. (C) EFS in CD3+CD4+/PD1+TIM3+ high and low expressing patients; (D) Competing risk analysis for EFS in PD1+TIM3+ high (>0.1% of CD4+T-cells) and low expressing patients.

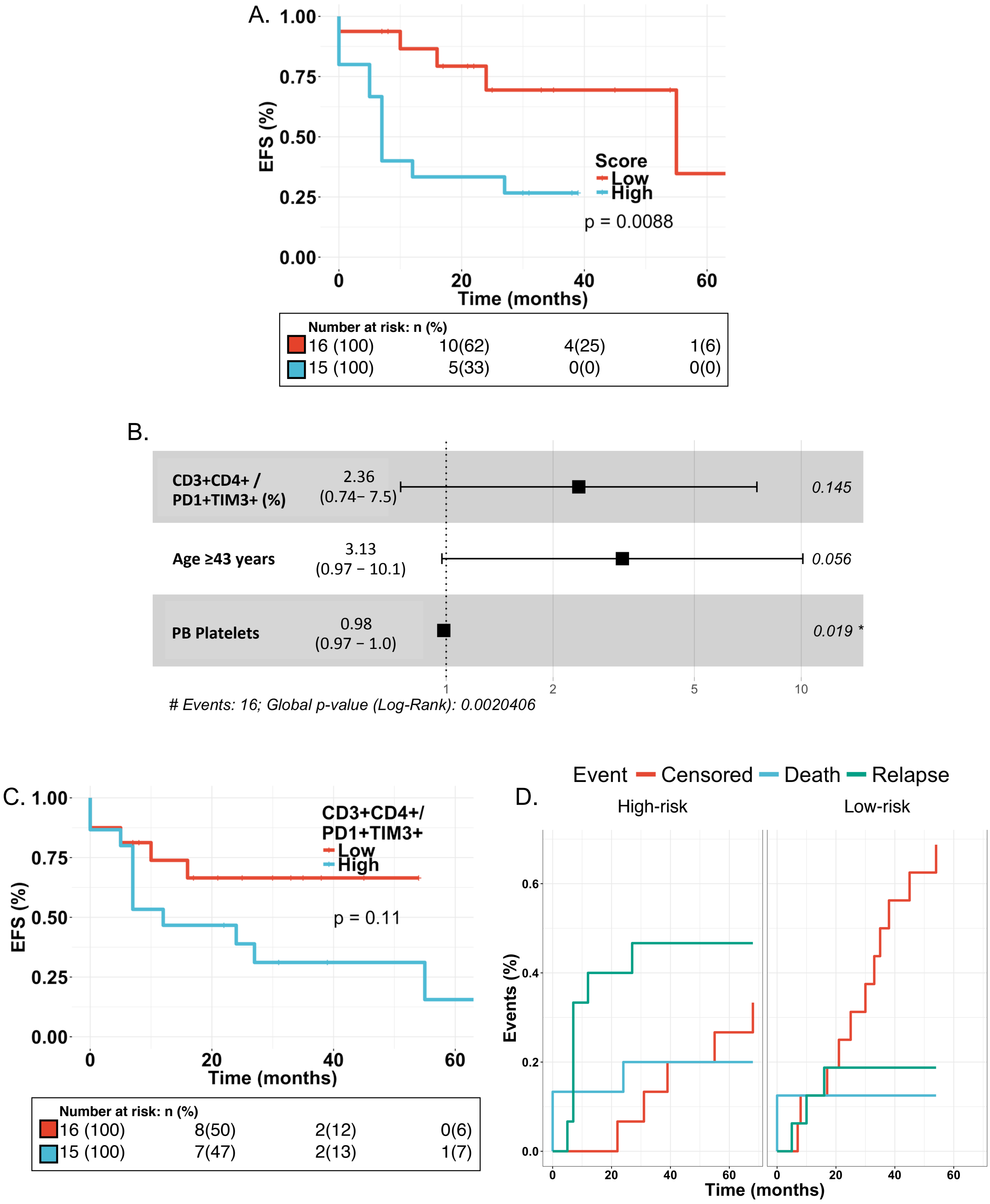

Supplementary Figure S4. Survival analysis in the Validation cohort. (A) The risk stratification model was divided by median into high and low-risk groups and plotted for event-free survival (EFS; Cox regression analysis, log-rank test). (B) Forest plot of the risk stratification model with continuous covariates. (C) EFS in CD3+CD4+/PD1+TIM3+ high and low expressing patients; (D) Competing risk analysis for EFS in PD1+TIM3+ high and low expressing patients.

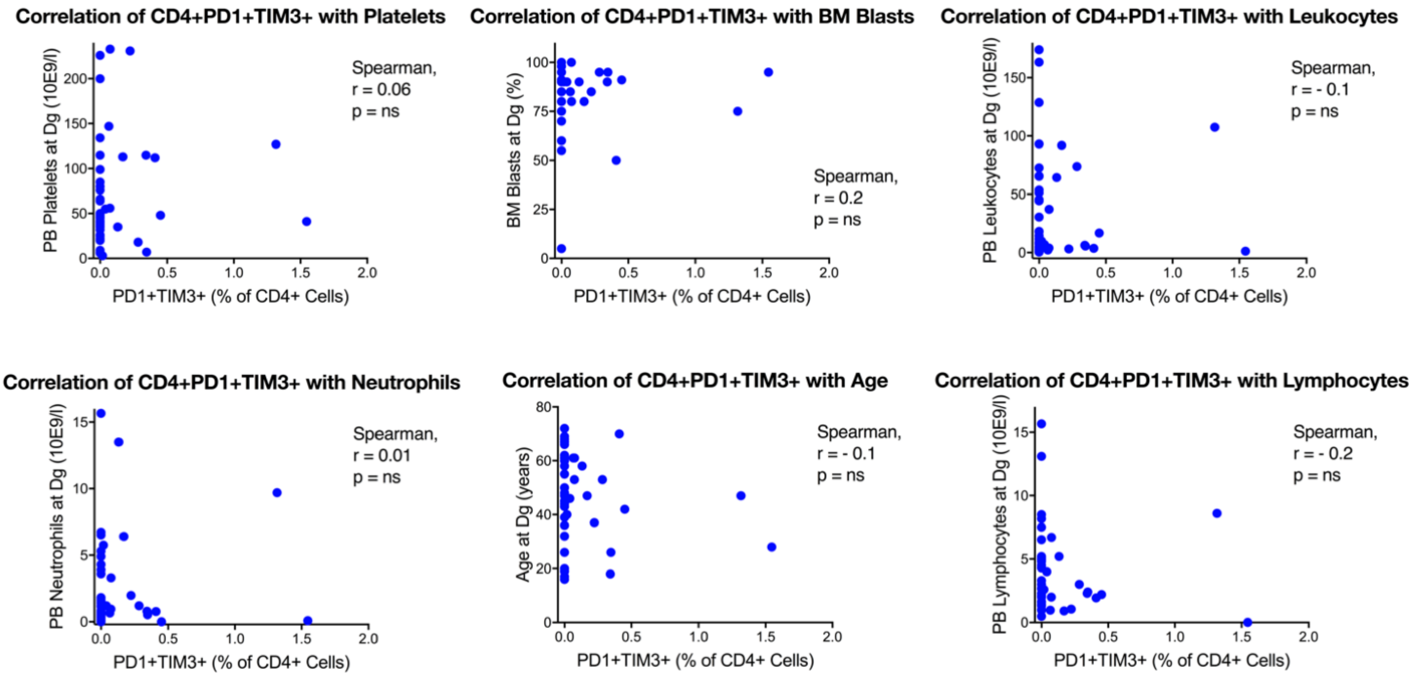

Supplementary Figure S5. Correlation (Spearman) of the proportion of CD4+ T-cells expressing PD1+TIM3+ (%) with diagnostic-phase peripheral blood (PB) platelet, leukocyte, neutrophil, and lymphocyte counts (10E/L), bone marrow (BM) blast proportion (%), and patient age.

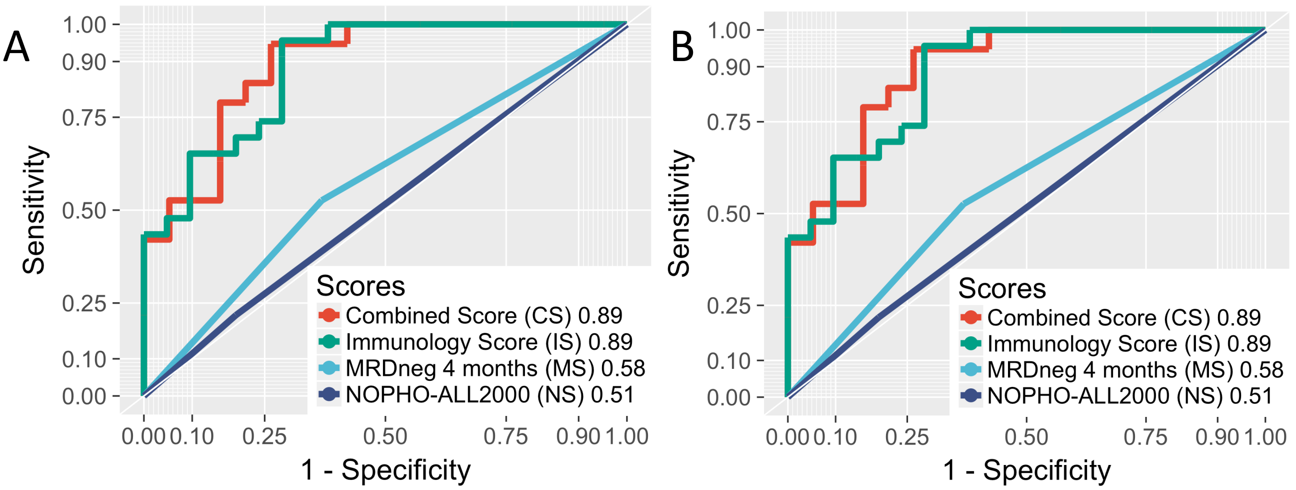

Comparison	CS vs MS	IS vs MS	CS vs NS	IS vs NS
P-value	***	***	***	***

Comparison	CS vs MS	IS vs MS	CS vs NS	IS vs NS
P-value	***	***	***	***

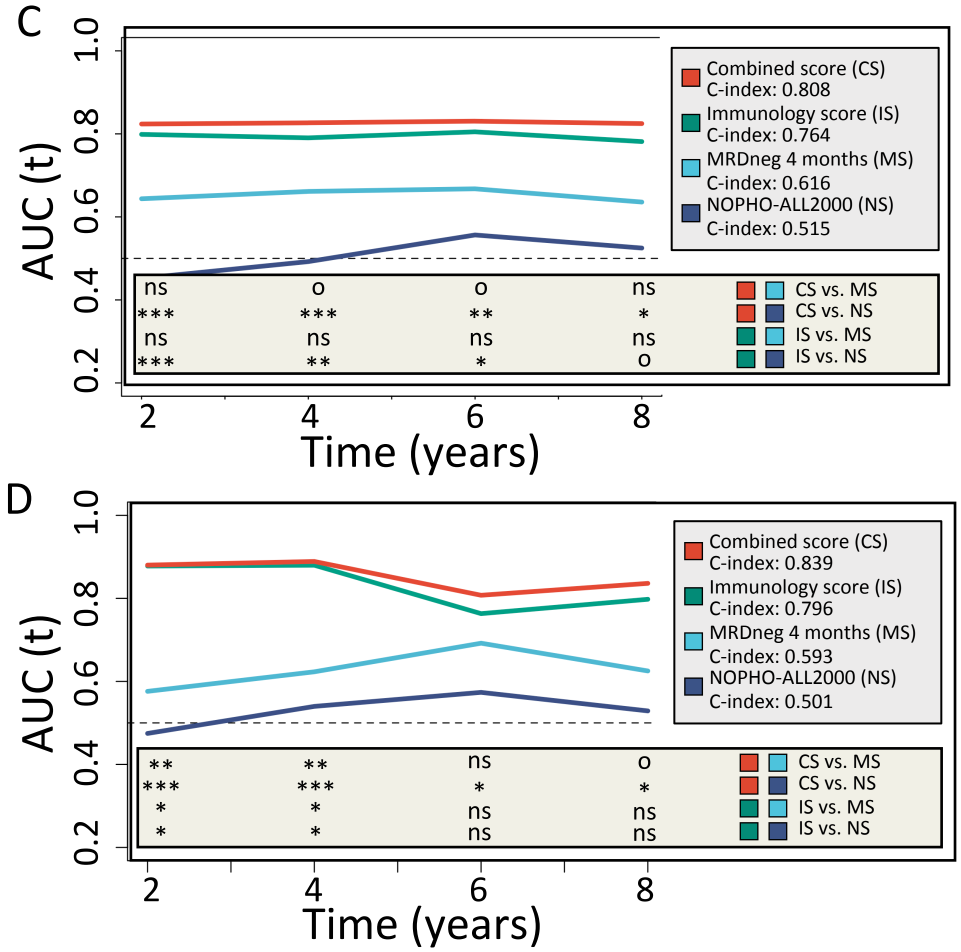

Supplementary Figure S6. To determine the prediction power of (A) Event-free survival (EFS) and (B) overall survival (OS), area under the receiver operating characteristic curves (AUROC) of our model (green line) was compared to stratification by MRD status at 4 months (light blue line) with the bootstrap method (number of iterations: 4000). Similarly, a combination of both models (red line) was developed and compared to stratification by MRD status at 4 months and original study protocol stratifications (dark blue line). Symbols for significance of the comparison: * p <0.05, ** p <0.01, *** p<0.001. The time-dependent receiver operating characteristic (ROC) curves (IPCW [inverse probability of censoring weighting] approach) and C-statistic values for (C) EFS, and (D) OS. Our prognostic model (green line) is compared to stratification by MRD status at 4 months post-diagnosis (light blue line). Their combined model (red line) is compared to stratification by MRD status at 4 months post-diagnosis and original study protocol stratifications (dark blue line). Significance is indicated at 2, 4, 6 and 8 years time points with following symbols: o p<0.10, * p<0.05, ** p<0.01, *** p<0.001.
